# Unequal Exposure to Kin Loss Across the Life Course in China

**DOI:** 10.1093/geroni/igaf122.255

**Published:** 2025-12-31

**Authors:** Jingwen Liu

**Affiliations:** Rice University, Houston, Texas, United States

## Abstract

Despite extensive research on the effects of specific bereavement events (e.g., parental or spousal loss) on health and well-being, few studies systematically examine how kin loss is structured across the life course and varies by social status, particularly in non-Western contexts. Applying nonparametric life table methods to the 2014 Life History Wave of the China Health and Retirement Longitudinal Study (CHARLS), this study investigates the cumulative risks and age-specific hazards of losing a mother, father, sibling, spouse, or child, respectively, in the full sample and by gender, education, and rural-urban hukou status. Descriptive analyses reveal that by 2014, 72.7% of respondents had lost a mother, 84.3% a father, 38.3% a sibling, 12.3% a spouse, and 11.9% a child. Kaplan-Meier survival estimates show that women are 1.1 to 1.5 times more likely than men to lose a child or spouse, with spousal loss risk peaking at 2.5 times between ages 65–70. Illiterate individuals are exposed to significantly higher risks of early parental deaths before midlife (peaking at 2-3 times), spousal (peaking at 3.2 times) and child deaths (peaking at 1.5 times) throughout adulthood, and sibling deaths (peaking at 2 times) across the whole life course. Rural residents are 1.5–2.1 times more likely to experience sibling and child loss, with child loss disparities persisting from ages 25 to 75. These findings highlight kin loss as an important but often overlooked determinant of social inequality, which may compound to further disadvantage the marginalized groups.

